# Spatio-temporal distribution of soil-transmitted helminth infections in Brazil

**DOI:** 10.1186/1756-3305-7-440

**Published:** 2014-09-18

**Authors:** Frédérique Chammartin, Luiz H Guimarães, Ronaldo GC Scholte, Mara E Bavia, Jürg Utzinger, Penelope Vounatsou

**Affiliations:** Department of Epidemiology and Public Health, Swiss Tropical and Public Health Institute, P.O. Box, CH-4002, Basel, Switzerland; University of Basel, P.O. Box, CH-4003, Basel, Switzerland; Immunology Service, Hospital Edgard Santos, Federal University of Bahia, Salvador, 40110-160 Bahia Brazil; Coordenação Geral de Hanseníase e Doenças em Eliminação, Secretaria de Vigilância em Saúde, Brasília, 70304-000 Distrito Federal Brazil; Preventive Medicine Department, Federal University of Bahia, Salvador, 40110-060 Bahia Brazil

**Keywords:** Soil-transmitted helminth, *Ascaris lumbricoides*, Hookworm, *Trichuris trichiura*, Predictive risk mapping, Bayesian geostatistics, Spatio-temporal model, Brazil

## Abstract

**Background:**

In Brazil, preventive chemotherapy targeting soil-transmitted helminthiasis is being scaled-up. Hence, spatially explicit estimates of infection risks providing information about the current situation are needed to guide interventions. Available high-resolution national model-based estimates either rely on analyses of data restricted to a given period of time, or on historical data collected over a longer period. While efforts have been made to take into account the spatial structure of the data in the modelling approach, little emphasis has been placed on the temporal dimension.

**Methods:**

We extracted georeferenced survey data on the prevalence of infection with soil-transmitted helminths (i.e. *Ascaris lumbricoides*, hookworm and *Trichuris trichiura*) in Brazil from the Global Neglected Tropical Diseases (GNTD) database. Selection of the most important predictors of infection risk was carried out using a Bayesian geostatistical approach and temporal models that address non-linearity and correlation of the explanatory variables. The spatial process was estimated through a predictive process approximation. Spatio-temporal models were built on the selected predictors with integrated nested Laplace approximation using stochastic partial differential equations.

**Results:**

Our models revealed that, over the past 20 years, the risk of soil-transmitted helminth infection has decreased in Brazil, mainly because of the reduction of *A. lumbricoides* and hookworm infections. From 2010 onwards, we estimate that the infection prevalences with *A. lumbricoides*, hookworm and *T. trichiura* are 3.6%, 1.7% and 1.4%, respectively. We also provide a map highlighting municipalities in need of preventive chemotherapy, based on a predicted soil-transmitted helminth infection risk in excess of 20%. The need for treatments in the school-aged population at the municipality level was estimated at 1.8 million doses of anthelminthic tablets per year.

**Conclusions:**

The analysis of the spatio-temporal aspect of the risk of infection with soil-transmitted helminths contributes to a better understanding of the evolution of risk over time. Risk estimates provide the soil-transmitted helminthiasis control programme in Brazil with useful benchmark information for prioritising and improving spatial and temporal targeting of interventions.

**Electronic supplementary material:**

The online version of this article (doi:10.1186/1756-3305-7-440) contains supplementary material, which is available to authorized users.

## Background

The nematode worms *Ascaris lumbricoides*, *Trichuris trichiura* and the two hookworm species *Ancylostoma duodenale* and *Necator americanus* are commonly referred to as soil-transmitted helminths [1]. These nematodes parasitise the human intestine and might lead to chronic infections with clinical consequences that undermine health of affected populations [1–3]. The World Health Organization (WHO) advocates a global control strategy against major helminthiases, emphasising preventive chemotherapy targeting high-risk communities, in combination with health education and sanitation improvement whenever resources allow [4].

Soil-transmitted helminthiases are of considerable public health concern in tropical and subtropical countries, where climatic conditions and poverty-related behaviours favour their transmission [5, 6]. South America is not spared [7, 8]. In Brazil, deworming campaigns were carried out covering up to 60% of the population but interventions have been interrupted in 2005 [4, 9], partially because of the decentralization of the programme [10]. Currently, WHO estimates that 9 million school-aged children in Brazil require preventive chemotherapy and anthelminthic administration of albendazole has been re-implemented in 2013 [4].

Spatial targeting of the population requiring preventive chemotherapy and other interventions is essential to implement tailored and cost-effective control measures. Bayesian geostatistical models are used to establish a statistical relationship between observed prevalence and environmental and socioeconomic risk factors, and predict the risk at unobserved locations, while accounting for spatial heterogeneity through spatially structured random effects [11]. These models have been widely applied to model soil-transmitted helminth risk at different scales [7, 12–14]. They are highly parameterised, and therefore estimation of model parameters relies on Markov chain Monte Carlo (MCMC) sampling methods. However, inference requires multiple inversions of the spatially structured variance-covariance matrix and MCMC methods are known to be computationally intensive. Thus, for large datasets, spatial process estimation can rely on low-rank approximation, such as the predictive process [7, 15].

By incorporating a temporal trend into the model, changes of the disease risk and pattern over time can be studied [7, 14, 16]. A temporal trend assumes that the infection risk changes over time by a certain amount, which is constant across space. However, the underlying latent spatial process might also vary over time. Bayesian formulations introduced by Knorr-Held [17] allow accounting for space-time interaction with an effect that is spatio-temporally structured through its precision matrix. Hence, such spatio-temporal models are able to estimate the spatial variation with time. The spatio-temporal aspect of helminthiases risk is an under-explored issue, mainly because of computational challenges in estimating highly parameterised models with MCMC algorithms. However, recent developments in Bayesian inference with integrated nested Laplace approximation (INLA) [18] using stochastic partial differential equations (SPDEs) [19] offer new opportunities for accurate fit of complex models at reasonable computational cost and time [20].

Here, we present an analysis of the spatio-temporal distribution of soil-transmitted helminth infection risks in Brazil. Our research extends a recent study that focused on the spatial distribution of soil-transmitted helminth infections in Brazil that was based on a relatively small database covering the period 2005-2009 [8]. We extended the survey period that now spans two decades (1995-2013) and focused on the space-time interactions of the disease patterns. We provide high-resolution spatial estimates of helminth species-specific infection risks and assess annualised deworming needs for school-aged children for Brazil. Historical data was extracted from the Global Neglected Tropical Diseases (GNTD) database [21], and Bayesian spatio-temporal models were fitted in a SPDEs/INLA framework. Predictors included in each model were selected within a Bayesian geostatistical variable selection approach that is well suited for large datasets.

## Methods

### Disease data

Prevalence survey data pertaining to *A. lumbricoides*, hookworm and *T. trichiura* in Brazil were extracted from the GNTD database (http://www.gntd.org). The GNTD database is an open-access platform gathering spatially explicit survey data on soil-transmitted helminthiasis and other neglected tropical diseases identified through systematic searches of readily available electronic databases and grey literature [21, 22]. The literature search for relevant soil-transmitted helminth prevalence data in Brazil was updated on 27th November 2013 and includes surveys conducted from 1995 onwards. The reader is referred to previous publications for further details on search strategy, geolocation and data quality appraisal [7, 21].

### Environmental, socioeconomic and population data

Table 1 summarises the sources and the spatial and temporal resolutions of environmental, socioeconomic and population data considered in our analysis. A total of 29 variables were taken into account as potential risk factors for soil-transmitted helminth infection. Environmental data included altitude, soil acidity, soil moisture and 19 bioclimatic variables related to temperature and precipitation. Socioeconomic proxies were: human development index (HDI), which is a measure of socioeconomic development based on life expectancy, education and income; human influence index (HII), which quantifies human influence on ecosystems; a poverty measure reflected by the percentage of people living with a household monthly income lower than US$ 60 (poor households); and a measure of rurality expressed by the percentage of rural households within municipalities. In addition, using census data we compiled the proportion of individuals within municipalities with access to improved water supply, sewage system and waste treatment. These last three variables were classified as improved according to the following criteria: (i) sewage system connected to a network or to a septic tank; (ii) water supply from a well or through the network; and (iii) waste collection by a cleaning service.

**Table 1:** **Data sources and properties of the predictors explored to model soil-transmitted helminth infection risk in Brazil**

Data type	Source	Temporal resolution	Spatial resolution
Temperature and precipitation^a^	WorldClim^b^	1950-2000	1 km
Altitude	SRTM^c^	2000	1 km
Soil acidity/soil moisture	ISRIC-WISE^d^	1960-2000	10 km
Human influence index (HII)	LTW^e^	2005	1 km
Human development index (HDI)	Atlas Brasil 2013^f^	2000 and 2010	Municipality
Poor households	Atlas Brasil 2013^f^	2000 and 2010	Municipality
Rurality, improved water supply, sewage system and waste treatment	Ministério da Saúde^g^	2010	Municipality
Population density	GPWFE^h^	2010	10 km

^a^A total of 19 climatic variables related to various factors were considered.

^b^WorldClim Global Climate database version 1.4; available at: http://www.worldclim.org/ (accessed: March 2012).

^c^Shuttle Radar Topography Mission (SRTM); available at: http://www.worldclim.org/ (accessed: March 2012).

^d^Global soil profile data ISRIC-WISE database version 1.2; available at: http://www.isric.org/ (accessed: December 2012).

^e^Last of the Wild Data version 2, 2005 (LTW-2): Global Human Footprint Dataset (Geographic). Wildlife Conservation (WCS) and Center for International Earth Science Information Network (CIESIN); available at: http://www.ciesin.org/wildareas/ (accessed: December 2013).

^f^Atlas do Desenvolvimento Humano no Brasil 2013; available at: http://atlasbrasil.org.br/ (accessed: December 2013).

^g^Ministério da Saúde, Brazil; available at: http://www2.datasus.gov.br/DATASUS/index.php (accessed: December 2013).

^h^Gridded population of the world: future estimates (GPWFE): Center for International Earth Science Information Network (CIESIN), UN Food and Agriculture Organization (FAO) and Centro Internacional de Agricultura Tropical (CIAT); available at: http://sedac.ciesin.columbia.edu/gpw (accessed: December, 2012).

Survey data were linked to potential risk factors based on their spatial proximity when they were available at fine spatial scale or according to their belonging to municipalities in case they were available at this resolution. Moreover, HDI and percentage of poor household data obtained in 2010 were assigned to prevalence data observed from 2005 onwards, while information obtained in 2000 was related to prevalence data prior to 2005.

### Statistical analysis

Soil-transmitted helminth infection prevalence data were modelled *via* binomial logistic regression with spatio-temporal random effects accounting for a latent spatial process varying with time. Exploratory analyses were carried out to assess correlations between potential predictors, as well as to explore their association with observed infection risks. Highly correlated potential risk factors (Pearson’s correlation coefficient >0.9) were grouped, with the aim to include not more than one of them in the models. Continuous predictors were standardised (by substracting their mean and dividing with standard deviation) to obtain estimates of the effects, which are comparable across the predictors.

Details on spatio-temporal model formulation and variable selection are given in the Additional file 1. In brief, risk factors included in the spatio-temporal models were selected through a Bayesian stochastic search variable selection approach [23]. We followed our previous procedure, which consists of selecting within a geostatistical framework the best predictors among highly correlated ones, while addressing non-linearity of the predictors [16]. We further extended this formulation in applications to large datasets, by estimating the spatial process through a predictive process approximation [15]. The inclusion of a variable in the model was defined as the product of two indicators: the first was assumed to be Bernoulli distributed and suggests the inclusion of the group of highly correlated variables, whilst the second followed a categorical prior distribution for selecting a single predictor within the group. In addition, regression coefficients were *a priori* parameterised with parameter expanded normal mixture of inverse-gamma (peNMIG) distributions [24], which ensure a rigorous selection of categorical variables. Models with the highest posterior probability identified the predictors to include in the final models.

Spatio-temporal distribution of soil-transmitted helminth infection risk was modelled using the methodology developed by Cameletti *et al.* [25] for spatio-temporal modelling. SPDEs were used to represent a Matérn spatio-temporal Gaussian field (GF) as a Gaussian Markov random field (GMRF), which in turn allowed an INLA algorithm to estimate model parameters. This approach provides considerable advantages in terms of computational cost compared to traditional MCMC algorithms. The spatio-temporal GF is characterised by a first-order autoregressive temporal effect and another temporally independent effect assumed to arise from a zero mean multivariate normal distribution with spatio-temporal covariance function of the Matérn family for identical time periods.

We further predict the risk of infection with individual soil-transmitted helminth species over a grid of 381,881 pixels (5 × 5 km spatial resolution). To validate our models, we re-fitted our spatio-temporal models on a randomly selected subset of approximately 80% of the data, and compared model-based estimated risks with the remaining 20% observed prevalences. Model predictive ability was measured by the proportion of correctly predicted values within the *k*th highest posterior density (HPD) interval with *k*% probability coverage of the posterior distribution varying from 50% to 95%. We used the mean error (ME) to assess the prediction bias.

### Population-adjusted risk and estimated treatment needs for school-aged children

The overall risk of soil-transmitted helminth infection was calculated for each of the samples of the predictive distribution, at each pixel, with a simple probabilistic model of combined infection divided by a factor of 1.06 [26]. To calculate population-adjusted risks, we multiplied predicted risks by the population at pixel level, summed them up over areas of interest, and divided them by the population of those areas.

Annualised treatment needs for school-aged children (age range: 5-14 years) for preventive chemotherapy were estimated by considering one treatment per year for children living in low-risk municipalities (population-adjusted risk between 20 and 50%) and two treatments for children living in high-risk areas (population-adjusted risk ≥50%), following WHO guidelines [27]. The school-aged population was estimated to represent 16.9% of the total population in Brazil, according to 2010 census data (http://www.ibge.gov.br/).

### Ethics statement

All data were obtained from existing databases without personal identifiers. Here, the data were further analysed to deepen our understanding of the spatio-temporal distribution of soil-transmitted helminth infections in Brazil. Hence, there were no specific ethical considerations for the current analysis.

## Results

**Figure 1:**
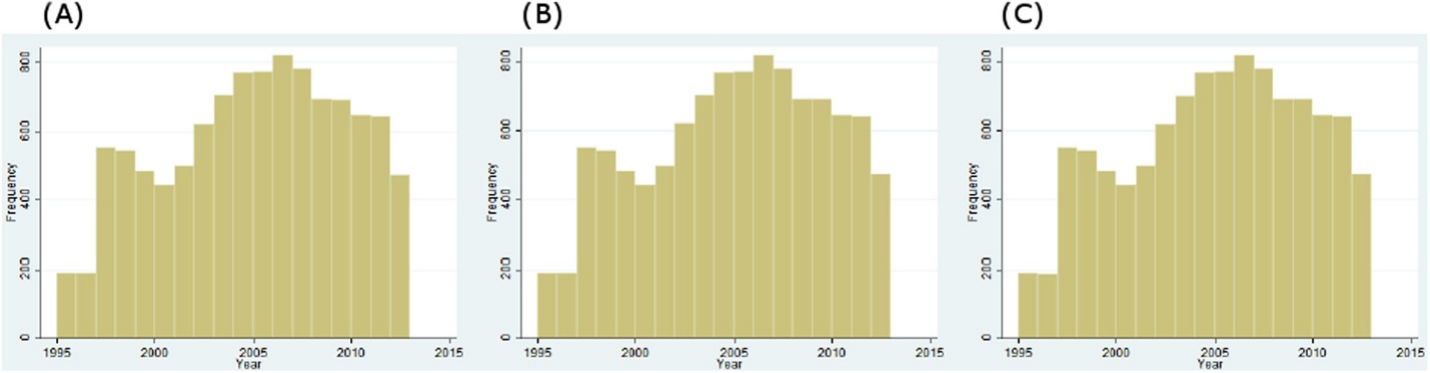
**Frequency distribution of soil-transmitted helminth survey data in Brazil from 1995 to 2013, stratified by year. (A)** *A. lumbricoides*, **(B)** *T. trichiura* and **(C)** hookworm.

**Figure 2:**
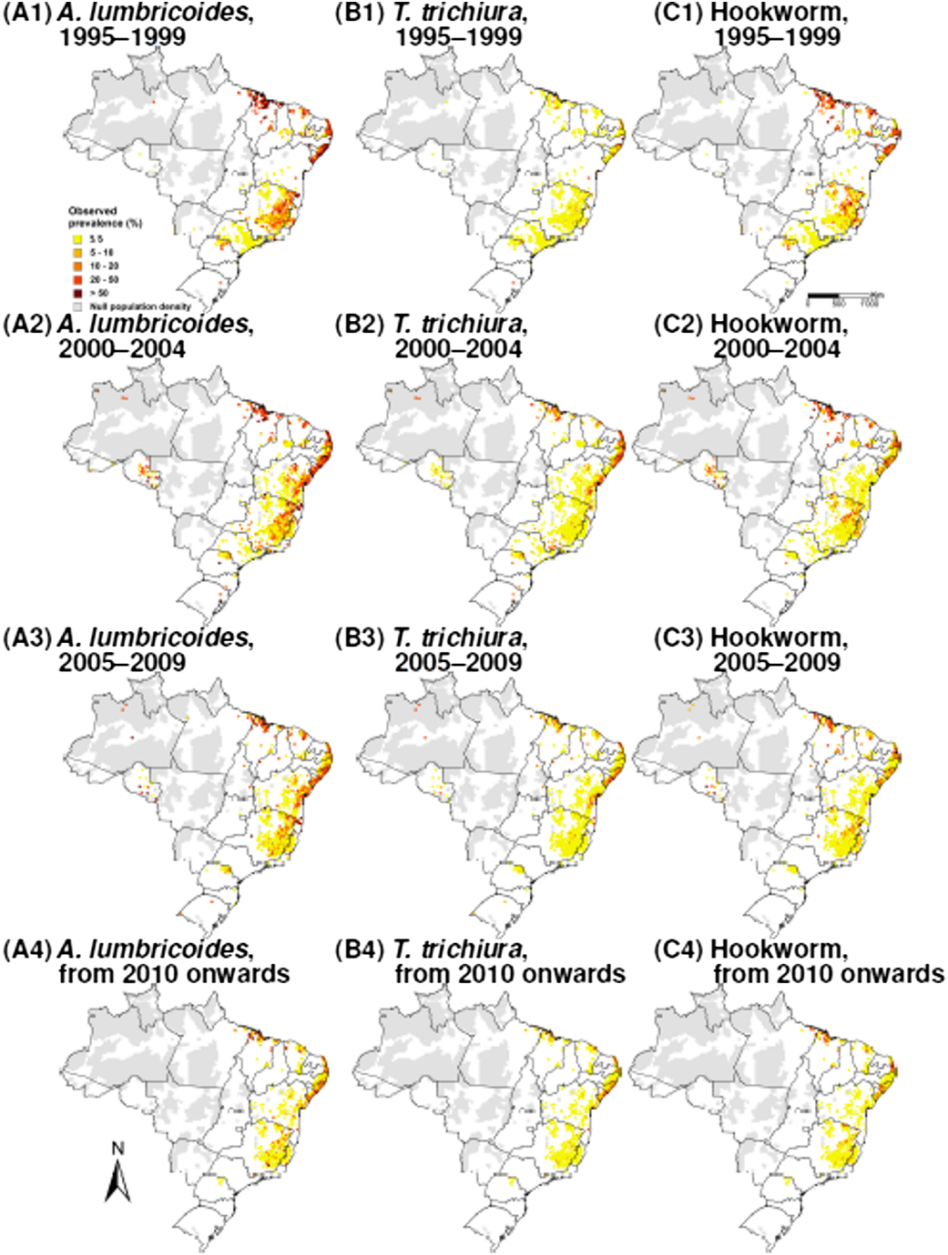
**Observed soil-transmitted helminth prevalence in Brazil, stratified by species and 5-year time periods. (A)** *A. lumbricoides*, **(B)** *T. trichiura* and **(C)** hookworm; (1) 1995-1999, (2) 2000-2004, (3) 2005-2009 and (4) from 2010 onwards.

**Figure 3:**
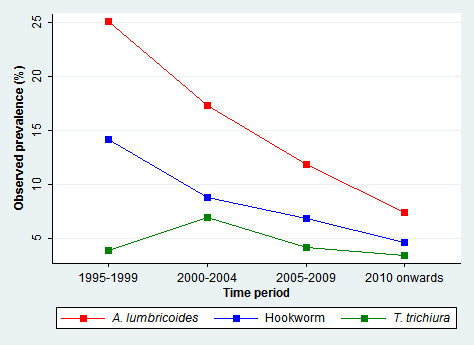
**Temporal trend and observed national prevalences for** ***A. lumbricoides*** **,** ***T. trichiura*** **and hookworm infections in Brazil.**

From 1995 onwards, we obtained spatially explicit information about prevalence of *A. lumbricoides*, *T. trichiura* and hookworm across Brazil for 10,513, 10,497 and 10,492 locations, respectively. The frequency distribution of individual soil-transmitted helminth species surveys, stratified by year, is depicted in Figure 1. The datasets included 1,587, 1,572 and 1,570 unique locations for *A. lumbricoides*, *T. trichiura* and hookworm, respectively. Data were aggregated over four time periods, i.e. (i) 1995-1999; (ii) 2000-2004; (iii) 2005-2009; and (iv) from 2010 onwards. Figure 2 shows the spatial distribution of the observed prevalence, stratified by soil-transmitted helminth species and time periods. As illustrated in Figure 3, a reduction of the overall raw prevalence was observed over the four periods, with the exception of *T. trichiura* infection, which showed peak prevalence in 2000-2004.

Results of the variable selection are given in Table 2. Out of the 29 potential predictors investigated, our variable selection procedure identified 14, 13 and 12 variables as being important for *T. trichiura*, *A. lumbricoides* and hookworm, respectively with model posterior probabilities of 93.5%, 44.8% and 25.3%. The selected variables were subsequently used to build spatio-temporal models.

**Table 2:** **Variables selected by a Bayesian variable selection approach applied within the geostatistical logistic regression model**

	***A. lumbricoides***infection	***T. trichiura***infection	Hookworm infection
**Group 1**			
Yearly mean temperature^a^	0	0	0
Maximum temperature of warmest month^a^	0	0	0
Minimum temperature of coldest month^a^	0	0	0
Mean temperature of wettest quarter	0	0	0
Mean temperature of driest quarter	0	0	0
Mean temperature of warmest quarter^a^	x	0	x
Mean temperature of coldest quarter^a^	0	x	0
**Group 2**			
Mean diurnal temperature range^b^	x	0	0
Yearly temperature range^a,b^	0	x	x
**Group 3**			
Isothermality	x	x	0
Temperature seasonality	0	0	x
**Group 4**			
Yearly precipitation^a^	x	0	0
Precipitation in wettest month	0	0	0
Precipitation in wettest quarter^a^	0	x	x
**Group 5**			
Precipitation in driest month^a,c^	0	x	0
Precipitation in driest quarter^c^	x	0	x
**Moderately correlated**			
Precipitation seasonality	x	x	x
Precipitation in warmest quarter^b^	x	x	x
Precipitation in coldest quarter^b,c^	x	x	x
Altitude	x	0	x
Soil moisture^a,b,c^	x	0	x
Soil pH^b,c^	x	x	x
Human development index (HDI)	x	x	x
Human influence index^b^ (HII)	0	x	0
Rural households^b,c^	0	x	0
Improved sanitation	0	0	0
Improved water supply^a,b,c^	0	0	0
Improved waste collection^b^	0	0	0
Poor households	x	x	0
Survey period	Fixed	Fixed	Fixed
**Posterior probability (%)**	44.8	93.5	25.3

^a^Categorised for *T. trichiura.*

^b^Categorised for hookworm.

^c^Categorised for *A. lumbricoides.*

x (selected), 0 (not selected).

The best model selected by the geostatistical variable selections is presented for each soil-transmitted helminth species, together with its posterior probability.

Parameter estimates of spatio-temporal multiple regression models, together with the ones of bivariate logistic associations with standard error clustered at location-level are presented for each soil-transmitted helminth species in Tables 3, 4 and 5. Results of bivariate logistic regressions show associations of the selected predictors with observed risk. Temperature and precipitation usually favour the risk of soil-transmitted helminthiasis, as reflected by the positive bivariate associations of temperature during warmest and coldest quarters and precipitation in coldest quarter and coldest month. However, precipitation during the warmest quarter was negatively associated with the risk of infection with any of the three soil-transmitted helminth species. Furthermore, important temperature and precipitation oscillations show a negative association with soil-transmitted helminth infection odds, as suggested by the effects of diurnal and yearly temperature ranges, low isothermality, as well as temperature and precipitation seasonality. The three infection risks were positively associated to proxies of poverty, as reflected by the positive effect of the percentage of poor households and the negative association of HDI.

**Table 3:** **Parameter estimates of bivariate and Bayesian spatio-temporal logistic models for** ***A. lumbricoides*** **infection risk in Brazil**

***A. lumbricoides***infection	Bivariate logistic ^†^	Spatio-temporal model
	OR (95% CI)	OR (95% BCI)
**Survey period**		
1995-1999	1.00	1.00
2000-2004	0.62 (0.56; 0.69)^*^	0.60 (0.51; 0.70)^*^
2005-2009	0.40 (0.34; 0.47)^*^	0.34 (0.28; 0.40)^*^
From 2010 onwards	0.24 (0.19; 0.30)^*^	0.14 (0.12; 0.17)^*^
**Mean temperature of warmest quarter**	1.91 (1.73; 2.12)^*^	0.98 (0.77; 1.24)
**Mean diurnal temperature range**	0.55 (0.50; 0.61)^*^	0.83 (0.73; 0.95)^*^
**Isothermality**	1.39 (1.25; 1.55)^*^	1.01 (0.90; 1.13)
**Yearly precipitation**	1.44 (1.32; 1.57)^*^	1.62 (1.43; 1.83)^*^
**Precipitation in driest quarter (mm)**		
<50	1.00	1.00
50-95	1.04 (0.75; 1.46)	1.56 (1.27; 1.92)^*^
≥95	1.83 (1.34; 2.51)^*^	1.36 (1.01; 1.84)^*^
**Precipitation seasonality**	0.88 (0.79; 0.97)^*^	0.92 (0.82; 1.02)
**Precipitation in warmest quarter**	0.53 (0.47; 0.59)^*^	0.65 (0.55; 0.76)^*^
**Precipitation in coldest quarter (mm)**		
<80	1.00	1.00
80-300	1.32 (0.98; 1.77)	0.65 (0.52; 0.81)^*^
≥300	4.21 (3.25; 5.45)^*^	0.92 (0.66; 1.30)
**Altitude**	0.49 (0.44; 0.55)^*^	0.82 (0.64; 1.06)
**Soil moisture (%)**		
<50	1.00	1.00
50-80	1.52 (1.21; 1.91)^*^	1.16 (0.96; 1.40)
≥80	0.92 (0.69; 1.22)^*^	1.01 (0.77; 1.33)
**Soil pH**		
<5.35	1.00	1.00
5.35-5.65	0.70 (0.54; 0.91)^*^	1.59 (1.36; 1.86)^*^
≥5.65	0.76 (0.59; 0.97)^*^	0.88 (0.74; 1.06)
**Human development index (HDI)**	0.62 (0.56; 0.69)^*^	1.29 (1.12; 1.49)^*^
**Poor households**	1.94 (0.68; 2.25)	1.81 (1.52; 2.15)^*^
		**Median (95% BCI)**
**Temporal autocorrelation**		0.03 (-0.02; 0.07)
**Spatial variance**		5.07 (4.73; 5.31)
**Spatial range (km)**		30.2 (28.1; 35.2)

^†^With standard error clustered at location level.

^*^Significant based on 95% CI or BCI.

OR: odds ratio; 95% CI: lower and upper bound of a 95% confidence interval; 95% BCI: lower and upper bound of a 95% Bayesian credible interval.

**Table 4:** **Parameter estimates of bivariate and Bayesian spatio-temporal logistic models for** ***T. trichiura*** **infection risk in Brazil**

***T. trichiura***infection	Bivariate logistic ^†^	Spatio-temporal model
	OR (95% CI)	OR (95% BCI)
**Survey period**		
1995-1999	1.00	1.00
2000-2004	1.83 (1.50; 2.23)^*^	3.47 (2.74; 4.40)^*^
2005-2009	1.05 (0.82; 1.36)	1.66 (1.30; 2.10)^*^
From 2010 onwards	0.87 (0.64; 1.17)	0.93 (0.71; 1.21)
**Yearly temperature range (°C)**		
<13	1.00	1.00
13-18	0.19 (0.15; 0.25)^*^	0.34 (0.25; 0.47)^*^
≥18	0.12 (0.09; 0.17)^*^	0.34 (0.22; 0.53)^*^
**Mean temperature of coldest quarter (°C)**		
<19	1.00	1.00
19-22	3.03 (2.25; 4.07)^*^	0.83 (0.61; 1.13)
≥22	7.97 (6.09; 10.42)^*^	1.42 (0.92; 2.18)
**Isothermality**	1.28 (1.15; 1.42)^*^	1.27 (1.09; 1.48)^*^
**Precipitation seasonality**	0.70 (0.64; 0.76)^*^	0.82 (0.68; 0.98)^*^
**Precipitation in wettest quarter (mm)**		
<560	1.00	1.00
560-680	1.03 (0.74; 1.45)	1.65 (1.22; 2.24)^*^
≥680	1.72 (1.24; 2.40)^*^	2.11 (1.48; 3.00)^*^
**Precipitation in warmest quarter (mm)**		
<250	1.00	1.00
250-440	0.62 (0.47; 0.83)^*^	1.50 (1.14; 1.99)^*^
≥440	0.18 (0.13; 0.24)^*^	2.45 (1.46; 4.11)^*^
**Precipitation in driest month (mm)**		
<14	1.00	1.00
14-16	1.07 (0.70; 1.64)	1.48 (1.10; 1.97)^*^
≥16	3.21 (2.16; 4.79)^*^	2.00 (1.32; 3.05)^*^
**Precipitation in coldest quarter (mm)**		
<80	1.00	1.00
80-300	2.04 (1.38; 3.02)^*^	1.13 (0.82; 1.55)
≥300	7.67 (5.48; 10.75)^*^	1.80 (1.11; 2.92)^*^
**Soil moisture (%)**		
<50	1.00	1.00
50-80	1.56 (1.15; 2.13)^*^	0.82 (0.61; 1.09)
≥80	0.91 (0.54; 1.52)	0.57 (0.38; 0.86)^*^
**Soil pH**		
<5.35	1.00	1.00
5.35-5.65	0.54 (0.38; 0.77)^*^	1.52 (1.20; 1.92)^*^
≥5.65	0.60 (0.45; 0.80)^*^	0.87 (0.67; 1.12)
**Human development index (HDI)**	0.74 (0.65; 0.84)^*^	1.45 (1.17; 1.79)^*^
**Human influence index (HII)**		
<20	1.00	1.00
20-26	1.39 (1.01; 1.91)^*^	1.49 (1.20; 1.86)^*^
≥26	2.14 (1.57; 2.91)^*^	1.86 (1.46; 2.37)^*^
**Rural households (%)**		
<25	1.00	1.00
25-50	1.01 (0.74; 1.39)	0.86 (0.68; 1.09)
≥50	0.61 (0.44; 0.74)^*^	0.66 (0.51; 0.85)^*^
**Poor households**	1.54 (1.27; 1.87)^*^	2.06 (1.58; 2.68)^*^
		**Median (95% BCI)**
**Temporal autocorrelation**		-0.05 (-0.10; 0.00)
**Spatial variance**		9.68 (9.27; 10.03)
**Spatial range (km)**		32.2 (29.9; 33.9)

^†^With standard error clustered at location level.

^*^Significant based on 95% CI or BCI.

OR: odds ratio; 95% CI: lower and upper bound of a 95% confidence interval; 95% BCI: lower and upper bound of a 95% Bayesian credible interval.

**Table 5:** **Parameter estimates of bivariate and Bayesian spatio-temporal logistic models for hookworm infection risk in Brazil**

Hookworm infection	Bivariate logistic ^†^	Spatio-temporal model
	OR (95% CI)	OR (95% BCI)
**Survey period**		
1995-1999	1.00	1.00
2000-2004	0.58 (0.50; 0.68)^*^	0.54 (0.43; 0.68)^*^
2005-2009	0.44 (0.36; 0.54)^*^	0.28 (0.22; 0.35)^*^
From 2010 onwards	0.29 (0.22; 0.39)^*^	0.13 (0.10; 0.17)^*^
**Mean temperature of warmest quarter**	2.00 (1.68; 2.37)^*^	1.50 (1.10; 2.05)^*^
**Temperature seasonality**	0.54 (0.43; 0.67)^*^	1.48 (1.21; 1.81)^*^
**Yearly temperature range (°C)**		
<13	1.00	1.00
13-18	0.61 (0.44; 0.86)^*^	0.98 (0.69; 1.37)
≥18	0.20 (0.15; 0.27)^*^	0.94 (0.60; 1.48)
**Precipitation in coldest quarter**	1.62 (1.43; 1.85)^*^	0.89 (0.69; 1.15)
**Precipitation in warmest quarter**	0.44 (0.35; 0.55)^*^	0.43 (0.33; 0.55)^*^
**Precipitation seasonality**	1.29 (1.15; 1.45)^*^	0.61 (0.49; 0.77)^*^
**Precipitation in driest quarter**	0.88 (0.76; 1.01)	0.74 (0.62; 0.87)^*^
**Precipitation in wettest quarter**	1.54 (1.31; 1.80)^*^	3.59 (2.79; 4.62)^*^
**Altitude**	0.56 (0.48; 0.67)^*^	0.99 (0.71; 1.37)
**Soil moisture (%)**		
<50	1.00	1.00
50-80	0.89 (0.64; 1.23)	0.52 (0.40; 0.67)^*^
≥80	0.49 (0.31; 0.78)^*^	0.30 (0.20; 0.43)^*^
**Soil pH**	0.87 (0.76; 1.00)	0.77 (0.70; 0.86)^*^
**Human development index (HDI)**	0.58 (0.53; 0.63)^*^	0.74 (0.68; 0.82)^*^
		**Median (95% BCI)**
**Temporal autocorrelation**		0.01 (-0.07; 0.06)
**Spatial variance**		8.92 (8.42; 9.43)
**Spatial range (km)**		29.7 (28.0; 31.4)

^†^With standard error clustered at location level.

^*^Significant based on 95% CI or BCI.

OR: odds ratio; 95% CI: lower and upper bound of a 95% confidence interval; 95% BCI: lower and upper bound of a 95% Bayesian credible interval.

In the spatio-temporal model, the odds of *A. lumbricoides* infection risk was positively associated with yearly precipitation, precipitation in driest quarter, soil pH (5.35-5.65), poor households and HDI, and negatively associated with mean diurnal temperature range, precipitation in warmest quarter and coldest quarter (80-300 mm).

For *T. trichiura*, the predictors with an important positive effect on the odds of the risk were: isothermality, precipitation in driest month, wettest, warmest and coldest quarters, soil pH (5.35-5.65), HDI, HII and poor households. On the other hand, the odds of *T. trichiura* infection were negatively associated with yearly temperature range, precipitation seasonality, soil moisture (≥80%) and rural households.

Hookworm infection odds increased with average temperature of warmest month, temperature seasonality, as well as with precipitation in the wettest quarter. On the other hand, precipitation in the warmest and driest quarter, precipitation seasonality, soil moisture, pH and HDI were negatively associated with the risk of hookworm infection.

The estimates of the effects of the survey periods indicate a decreasing trend for both *A. lumbricoides* and hookworm infection risks in the period studied, i.e. from 1995 onwards until late 2013. For *T. trichiura*, there was no important effect of survey period after 2010 compared to the preceding decade.

**Figure 4:**
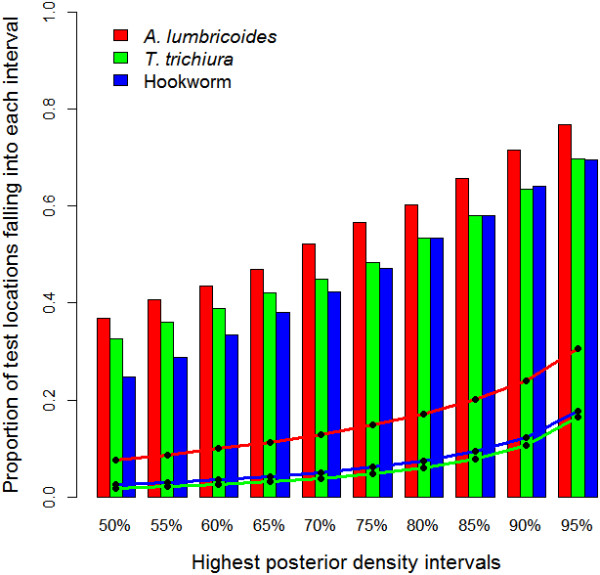
**Model validation results.** Proportion of surveys with prevalence of infection falling in the predicted highest posterior density (HPD) intervals (bar plots) for *A. lumbricoides*, *T. trichiura* and hookworm. The line plots show the corresponding width of the predicted HPD region.

Figure 4 shows the results of model validation. The risks of soil-transmitted helminth infection were correctly predicted within a 95% credible interval for 77% of the tested data for *A. lumbricoides*, 70% for *T. trichiura* and 69% for hookworm. The ME was -3.03%, -2.26% and -2.75% for the three species, respectively, suggesting that our models slightly underestimate the observed prevalences.

**Figure 5:**
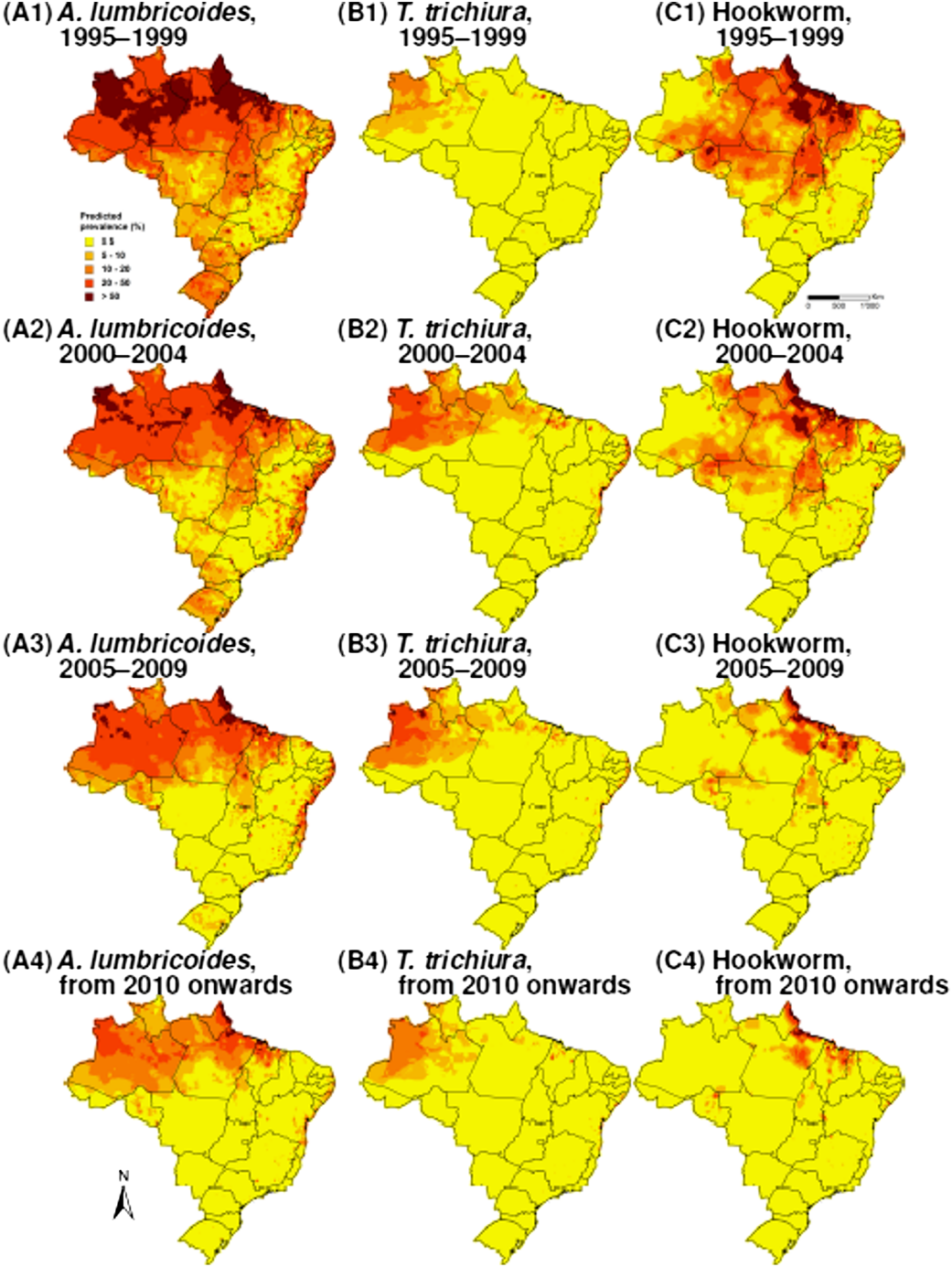
**Predicted soil-transmitted helminth risk in Brazil, stratified by species and 5-year time periods. (A)** *A. lumbricoides*, **(B)** *T. trichiura* and **(C)** hookworm; (1) 1995-1999, (2) 2000-2004, (3) 2005-2009 and (4) from 2010 onwards.

Model-based predictions of the geographical distribution of the three soil-transmitted helminth species considered in our analyses are presented in Figure 5, for each of the four time periods. From 2010 onwards, *A. lumbricoides* infection presents larger risk areas compared to the other two species, with higher risk in the northern part of the country. The highest risk for *T. trichiura* was found in the north-western part of Brazil, while the risk for hookworm was higher along the northern coast. Our maps also highlight the temporal evolution of the risk for an infection with any of these three soil-transmitted helminth species over the past 20 years. Apparent shrinkage of high-risk areas was observed for *A. lumbricoides* and hookworm. Spatial correlation was estimated around 30 km for each of the three soil-transmitted helminth species, and spatial variance extended from 5.07 to 9.68. Temporal autocorrelation was generally weak, suggesting that temporal structure was explained by the temporal trend, as well as by changes in HDI and percentage of poor households over time.

**Figure 6:**
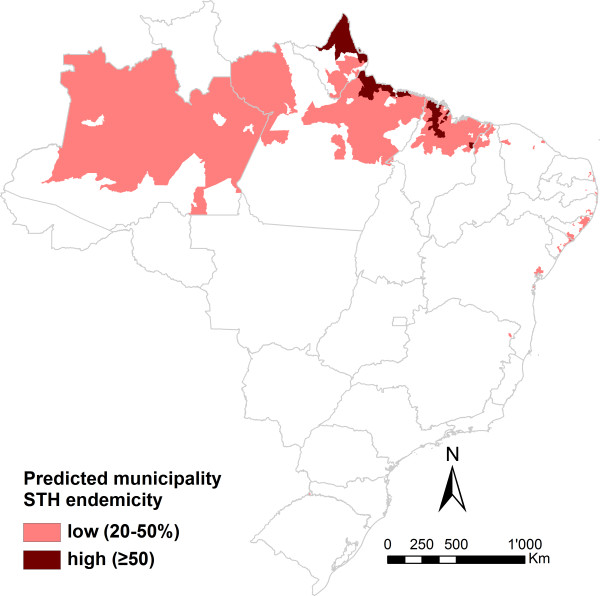
**Estimated soil-transmitted helminthiasis (STH) endemicity of the Brazilian municipalities for intervention planning according to WHO guidelines pertaining to preventive chemotherapy.**

Predicted population-adjusted risk estimates in Brazil are given for each survey period analysed (Table 6). Based on predictions from 2010 onwards, we estimated that 10.9 million people were infected with soil-transmitted helminths in Brazil (population-adjusted risk = 6.0%; 95% Bayesian credible interval (BCI): 5.4-6.9%). Single species infection population-adjusted risks were estimated at 3.6% for *A. lumbricoides* (95% BCI: 3.0-4.3%), 1.7% for hookworm (95% BCI: 1.4-2.3%), and 1.4% for *T. trichiura* (95% BCI: 1.1-1.7%). Low-risk (population-adjusted risk 20-50%) and high-risk (population-adjusted risk ≥50%) municipalities are highlighted in Figure 6. The highest population-adjusted risk of soil-transmitted helminthiasis was found along the northern coast. We estimated that 1.8 million doses of anthelminthic treatments are required for preventive chemotherapy targeting school-aged children at municipality level in Brazil.

**Table 6:** **Predicted population-adjusted risk of** ***A. lumbricoides*** **,** ***T. trichiura*** **, hookworm and overall soil-transmitted helminth infection in Brazil, stratified by survey period**

Survey period	***A. lumbricoides***	***T. trichiura***	Hookworm	Soil-transmitted helminth
	infection risk (%)	infection risk (%)	infection risk (%)	infection risk (%)
1995-1999	15.6 (13.6; 18.0)	1.8 (1.4; 2.3)	7.6 (6.6; 9.0)	20.9 (19.0; 23.2)
2000-2004	11.4 (10.0; 13.0)	4.5 (3.6; 5.7)	5.7 (4.9; 6.9)	17.9 (16.5; 19.7)
2005-2009	7.9 (6.8; 9.1)	2.5 (2.1; 3.2)	2.8 (2.4; 3.4)	11.5 (10.4; 12.6)
From 2010 onwards	3.6 (3.0; 4.3)	1.4 (1.1; 1.7)	1.7 (1.4; 2.3)	6.0 (5.4; 6.9)

Population-adjusted risks are given with their 95% Bayesian credible interval (BCI).

Risk is adjusted on population of 2010 for survey periods from 2005 onwards and on population of 2000 for survey periods prior to 2005.

## Discussion

The current study focuses on the spatio-temporal distribution of *A. lumbricoides*, hookworm and *T. trichiura* risk in Brazil and therefore complements and expands upon a recent study that investigated spatial patterns [8]. We present predictive risk maps at high spatial resolution from 1995 onwards at 5-year increments. Additionally, we provide a map that highlights municipalities that require preventive chemotherapy targeting school-aged children according to recommendations put forward by WHO [4]. Our analyses provide new insight into spatio-temporal risk profiling of helminthiasis based on a large ensemble of geolocated survey data by considering space-time interactions.

We provide model-based evidence of a decrease of *A. lumbricoides* and hookworm infection risks over the past 20 years in Brazil. Interestingly, the temporal evolution of the third common soil-transmitted helminth species – *T. trichiura* – increased after 2000, started to decline from 2005 onwards, and finally reached similar levels to the situation in 1995-1999 from 2010 onwards. We believe that the main reasons explaining the lower risk of *A. lumbricoides* and hookworm from 2010 onwards compared to the situation 20 years ago are the social and economic development, coupled with deworming activities. Nevertheless, it is important to note that no mass deworming activities were carried out by the Ministry of Health (MoH) in Brazil from 2005 to 2011 [4, 9]. The question arises why a similar decline was not observed for *T. trichiura*. Differences may reflect differential efficacies of the widely used deworming drugs albendazole and mebendazole. While both drugs show high cure and egg reduction rates against *A. lumbricoides*, and albendazole shows satisfactory efficacy against hookworm, neither drug results in high efficacy against *T. trichiura* [28, 29]. These differences might explain the delayed risk change profile for *T. trichiura*.

Our predictive risk maps highlight that high-risk areas of *A. lumbricoides* and *T. trichiura* infections occur in the north-western part and along the eastern coast of Brazil, while high-risk areas of hookworm infection is concentrated along the northern coast. This is coherent with patterns highlighted by two previous analyses [7, 8]. However, our population-adjusted estimates for the period 2005-2009 of 7.9% for *A. lumbricoides*, 2.5% for *T. trichiura* and 2.8% for hookworm are smaller than those based on predictions from 2005 onwards stemming from a spatial analysis of South America (i.e. 14.3% for *A. lumbricoides*, 10.1% for *T. trichiura* and 12.3% for hookworm) [7]. These differences might be explained by the inclusion of socioeconomic factors in the current analysis. Our previous work did not include poverty indicators due to the difficulty of deriving consistent measures among different countries. In comparison to a temporal trend included as a covariate, which indicates the change of magnitude of the risk over time [7, 14], spatio-temporal models, as developed in this analysis, highlight the changes in the geographical patterns of risk over time. Hence, our analysis highlights the importance of considering the temporal aspect of infection risk, especially in a country like Brazil, where socioeconomic conditions have considerably improved and infectious diseases risk has declined over time [7, 30]. In comparison to Scholte *et al*. [8], who analysed a restricted dataset with data provided by the national schistosomiasis control programme for the period 2005-2009, we estimated considerably smaller risks for both *A. lumbricoides* (7.9% *versus* 15.6%) and *T. trichiura* (2.5% *versus* 10.1%). We explain these differences by a considerably higher spatial coverage of our data. Recently, Pullan and colleagues [6] estimated the global risk of soil-transmitted helminth infections for the year 2010 based on empirical approaches, which do not account for small-scale spatial variation. For Brazil, they estimated a risk between 1% and 10% for *T. trichiura* and hookworm infections, which is comparable to our estimates of 1.4% and 1.7% from 2010 onwards. However, we estimated a risk of 3.6% for *A. lumbricoides*, whilst Pullan *et al.* [6] estimates a risk between 10% and 20%. This difference highlights the importance of capturing small-scale variation in estimating the risk of helminth infection and other neglected tropical diseases.

Parameter estimates of the spatio-temporal models reflect the climatic suitability and socioeconomic conditions that favour soil-transmitted helminthiasis transmission in Brazil. Each soil-transmitted helminth species risk is influenced by complex interactions of the predictors selected by our variable selection approach. In particular, our analysis confirms that warm and humid conditions are suitable for soil-transmitted helminth egg and larval development [31, 32]. Positive associations of precipitation were observed for the three soil-transmitted helminth species and temperature was an important risk factor for hookworm. Extreme weather conditions could adversely affect development and survival of helminthic free-living stages. Indeed, larvae optimally hatch within certain temperature limits [33], suggesting that extreme temperatures might impair their development. Larger range of temperature during the day showed a negative effect in *T. trichiura* and *A. lumbricoides* models, while strong isothermality positively impacts the risk of *T. trichiura*, confirming this hypothesis. Furthermore, it has been speculated that heavy rainfall might wash out soil-transmitted helminth eggs from the soil [7, 34, 35]. The negative effects of precipitation seasonality in *T. trichiura* and hookworm models, precipitation in warmest quarter in *A. lumbricoides* model, as well as of soil moisture in hookworm model point in that direction. We also note that high isothermality, low range of temperature during the day and low precipitation seasonality are typical characteristics of northern equatorial and tropical humid regions of Brazil, suggesting that those climatic areas are suitable for transmission. The optimal soil acidity for *A. lumbricoides* and *T. trichiura* transmission ranges between pH values of 5.35 and 5.65, however, hookworm prefers somewhat less acid conditions.

Our analysis also highlights the intimate connection of soil-transmitted helminth infection with poverty. Indeed, high percentage of poor households was an important risk factor for both *T. trichiura* and *A. lumbricoides* infections, after accounting for HDI. Poor households generally show lower frequencies of access and use of clean water and improved sanitation and thus are at higher odds of soil-transmitted helminth infection [36, 37]. Another interesting aspect is the positive effect of HII and low percentage of rural households associated with *T. trichiura* risk, confirming previous findings [5, 7]. These observations suggest that *T. trichiura* infection might be more prevalent in urban compared to rural settings [5].

Most of the soil-transmitted helminth data (97.5%) stem from the Brazilian schistosomiasis control programme, which took advantage of the Kato-Katz technique, which allows concurrent diagnosis of soil-transmitted helminths, while screening for *Schistosoma mansoni* eggs in faecal thick smears. Brazil launched its national schistosomiasis control programme in 1975 with the aim of reducing schistosomiasis-related morbidity. Regarding soil-transmitted helminthiasis, the MoH re-started a mass deworming campaign for school-aged children in 2013, prioritising areas characterised by a low HDI. This campaign will now be extended to the whole of Brazil. Data generated by this programme will facilitate the study of the evolution of the risks and evaluation of the impact of interventions. It will be important to specifically inform about the situation regarding the population targeted by the interventions (e.g. school-aged children or entire communities). Data that we analysed in the current study were mainly collected within the whole population (only 1% referred to children exclusively). Thus, we might underestimate the risk among children, as it is known that they are usually at a higher risk of soil-transmitted helminth infections, particularly *A. lumbricoides* and *T. trichiura* [38]. Importantly though, despite low HDI showing a positive association with the three soil-transmitted helminth infection risks in bivariate associations, our spatio-temporal analysis indicates that helminthiasis risk was driven by complex environmental/socioeconomic interactions. Hence, we believe that our estimates provide useful information for a refined target of interventions.

From a modelling point of view, it is important to highlight that SPDE methodology and INLA enabled us to fit our spatio-temporal models at a reasonable computational cost (around 4 hours per model, including fitting and prediction). Implementing this type of model would have been difficult to achieve with MCMC, especially because of convergence problems and large number of locations for spatio-temporal process estimation. With regard to the risk of the three soil-transmitted helminth species, residual spatial correlation was moderate (around 30 km) and temporal autocorrelation was weak. Hence, most of the spatial and temporal dynamics were captured by the covariates of our models.

## Conclusions

The methodology employed in the current analysis enables fitting more complex models and provides a useful tool for joint analysis of space and time components for risk profiling. The analysis of the spatio-temporal aspect of the risk of soil-transmitted helminth infections deepens our understanding of the evolution of the risk across time and enables more accurate predictions of the infection risks. We hope that our estimates will provide useful benchmark information for the soil-transmitted helminthiasis control programme in Brazil to prioritise interventions and enhance spatial targeting.

## Electronic supplementary material

Additional file 1: **Geostatistical variable selection and Bayesian spatio-temporal model formulations.** (PDF 171 KB)

## Abbreviations

BCI: Bayesian credible interval
GF: Gaussian field
GMRF: Gaussian Markov random field
GNTD: Global Neglected Tropical Diseases (database)
HDI: Human development index
HII: Human influence index
HPD: Highest posterior density
INLA: Integrated nested Laplace approximation
MCMC: Markov chain Monte Carlo
ME: Mean error
MoH: Ministry of Health
peNMIG: Parameter expanded normal mixture of inverse-gamma
SPDE: Stochastic partial differential equation
WHO: World Health Organization.
