# Supplementary material for: Spatio-temporal distribution of soil-transmitted helminth infections in Brazil
Source: Parasit Vectors. 2014 Sep 18;7:440. doi: 10.1186/1756-3305-7-440 (PMC4262198; doi:10.1186/1756-3305-7-440)
Supplement: Supplementary file 1 — Additional file 1: Geostatistical variable selection and Bayesian spatio-temporal model formulations. (PDF 171 KB) [file 13071_2014_1618_MOESM1_ESM.pdf]

Additional file 1

Geostatistical variable selection formulation

Let Y_i, n_i and p_i be the number of infected individuals, the number of individuals screened, and the prevalence of infection at location i $(i = 1, ..., N)$. We assume that Y_i arises from a Binomial distribution, i. e., $Y_i \sim Bn(n_i, p_i)$. Potential predictors $X_{j_b}^{(b)}$, $j_b = 1, ..., J_b$ are divided into B groups b, $(b = 1, ..., B)$, where $B-1$ groups contain predictors wich are considered highly correlated with a Pearson coefficient > 0.9, while the Bth group includes predictors that exhibit only moderate correlation with other potential predictors. In addition, potential predictors presenting a non-linear association to the infection risk in explanatory analyses have been categorised and we define $X_{lj_b}^{(b)}$ as being the lth categorical form of predictor $X_{j_b}^{(b)}$, where $l = 1, ..., L$ categories (excluding baseline). Our variable selection procedure aims to select most important predictors, while accounting for spatial correlation, addressing non-linearity of the predictors, and forcing the model to choose a maximum of one predictor among the ones considered as highly correlated. To that end, we model a categorical temporal trend T_{il} $(l = 1, ..., L$ categories), the potential predictors $X_{lj_bi}^{(b)}$ and a spatial random effect φ_i on the logit scale, such as :

$$logit(p_i) = \beta_0 + \sum_{l=1}^{L} T_{il}\beta_{1l} + \sum_{b=1}^{B}\sum_{j_b=1}^{J_b} \alpha_{j_b} \sum_{l=1}^{L_{j_b}} \xi_{lj_b} X_{lj_bi}^{(b)} + \varphi_i,$$

where regression coefficients of potential predictors X_{j_b} are defined as the product of an overall contribution α_{j_b} and the effect ξ_{lj_b} of each of its elements (i. e., categories).

Within a Bayesian framework of inference, we assign a spike and slab prior (Scheipl et al., 2012; Chammartin et al., 2013a,b) to α_{j_b}, which is a scaled normal mixture of inverse-gamma, that is $\alpha_{j_b} \sim N(0, \tau_{j_b}^2)$, where $\tau_{j_b}^2 \sim \gamma_1^{(b)}\gamma_{2j_b}^{(b)} IG(a_\tau, b_\tau) + (1-\gamma_1^{(b)}\gamma_{2j_b}^{(b)})\upsilon_0 IG(a_\tau, b_\tau)$, where a_τ and b_τ are fixed parameters of non-informative inverse-gamma distribution set to 5 and 25, respectively, while υ_0 is a small constant set to 0.00025, shrinking α_{j_b} to zero when the predictor is excluded. The product of the two indicators $\gamma_1^{(b)}$ and $\underline{\gamma}_2^{(b)} = (\gamma_{21}^{(b)}, ..., \gamma_{2J_b}^{(b)})^T$ indicates the presence or absence of the predictors in the model. In particular, $\gamma_1^{(b)}$ determines the presence or absence of the group b in the model and $\gamma_{2j_b}^{(b)}$, allows selection of a single predictor within the group. A Bernoulli and a categorical prior distribution are assigned to $\gamma_1^{(b)}$ and $\underline{\gamma}_2^{(b)}$, respectively, such as $\gamma_1^{(b)} \sim Bern(\Omega_1^{(b)})$ and $\underline{\gamma}_2^{(b)} \sim Cat(J_b, \Omega_{21}^{(b)}, ..., \Omega_{2J_b}^{(b)})$ with inclusion probabilities $\Omega_1^{(b)}$ and $\underline{\Omega}_2^{(b)}$. To allow greater flexibility in estimating model size, these probabilities are considered as hyper-parameters having non-informative beta and Dirichlet distributions ; $\Omega_1^{(b)} \sim Beta(1,1)$, $\underline{\Omega}_2^{(b)} = (\Omega_{21}^{(b)}, ..., \Omega_{2J_b}^{(b)})^T \sim Dirichlet(1, ..., 1)$. A mixture of two Gaussian distributions is assumed for ξ_{lj_b}, $\xi_{lj_b} \sim N(m_{lj_b}, 1)$, $m_{lj_b} \sim 1/2\delta_1(m_{lj_b}) + 1/2\delta_{-1}(m_{lj_b})$, which shrinks ξ_{lj_b} towards $|1|$ (multiplicative identity). For predictors moderately correlated, γ_{2j_b} is fixed to 1, while the effect of linear predictors is only defined by an overall contribution of α. In addition, non-informative normal priors have been assigned to the constant β_0 and the effects β_{1l} of the temporal trend ; $\beta_0, \beta_{1l} \sim N(0, 100)$.

Large matrix computation cost in estimating this latent spatial process φ is overcome with the predictive process estimation (Banerjee et al., 2008). In more details, φ is estimated from a subset of 200 locations (knots) $\{s_k^*, k = 1, .., K\}$ with latent observations $\varphi^* = (\varphi_1^*, ..., \varphi_K^*)^T$, $\varphi^* \sim MVN(0, \Sigma^*)$. Σ^* is the KXK variance-covariance matrix modelled by an isotropic exponential correlation function of distance, i. e, $\Sigma_{cd}^* = \sigma_{sp}^2 exp(-\rho d_{cd})$, where d_{cd} is the Euclidean distance between locations c and d, σ_{sp}^2 is the geographical variability, and ρ controls the rate of correlation decay. Inverse gamma distribution $\sigma_{sp}^2 \sim IG(2.01, 1.01)$ is chosen for the variance σ_{sp}^2 and a gamma distribution is assumed for the spatial decay ρ, $\rho \sim G(0.01, 0.01)$. Spatial random effect φ at original set of locations are predicted via the conditional mean $Q^T\Sigma^{*-1}\varphi^*$, where $Q = Cov(\varphi^*, \varphi)$ is a NXK matrix of the covariance function between the K knots and the N observed locations. Minimax space filling sampling (Johnson et al., 1990; Diggle and Lophaven, 2006) is used to select the knots using the cover.design routine in R (The R Foundation for Statistical Computing R v.3.0.2).

Geostatistical variable selection was run in JAGS through the rjags library of R (The R Foundation for Statistical Computing v.3.0.2) in JAGS 3.4.0 with on chain sampler and 40,000 iterations (including a burn-in of 10,000 iterations). Final 10,000 iterations were used to calculate models posterior probabilities

and the subset of variables included in the models with the highest posterior probabilities identified the final models.

Bayesian spatio-temporal model formulation

Our Bayesian spatio-temporal model formulation follows the approach introduced by Cameletti et al. (2013). In particular, we define Y_{it}, p_{it} and n_{it} as the number of infected individuals, the number of individuals screened, and the prevalence of infection at location i ($i = 1, ..., N$) for time t ($t = 1, ..., T$), and we assume Y_{it} to be generated from a binomial distribution, i.e., $Y_{it} \sim Bin(p_{it}, n_{it})$. Prevalence of infection is then linearly regressed on the logit scale as follows : $logit(p_{it}) = X_{it}^T \underline{\beta} + \varphi_{it}$, where X is the matrix of explanatory variables (including an intercept, a temporal trend, and the predictors selected by the variable selection), $\underline{\beta}$ is the regression coefficient vector, and $\underline{\varphi}$ is a spatio-temporally-structured random effect. We allow the spatio-temporal process φ_{it} to change in time with a first order autoregressive process (AR1), such as :

$$\begin{array}{ll} \varphi_{it} = \omega_{i1} & \text{if } t = 1 \\ \varphi_{i,t} = a\varphi_{i,t-1} + \omega_{i,t} & \text{if } t = 2, .., T, \end{array}$$

with a temporal autoregressive coefficient a, $|a| < 1$ and a temporally independent spatially-structured effect ω which is assumed to be multivariate normal with zero mean and spatio-temporal covariance function of the Matérn family :

$$Cov(\omega_{i,t}, \omega_{j,t'}) = \left\{ \begin{array}{ll} 0 & \text{if } t \neq t' \\ \sigma_\omega^2 C(d_{ij}) & \text{if } t = t' \end{array} \right. for\, i \neq j,$$

where σ_ω^2 is the variance of the structured effect ω, (σ_ω^2=Var($\omega_{i,t}$)). The spatial correlation function $C(d_{ij})$ is function of the Euclidean distance between locations i and j (d_{ij}) and is defined by the Matérn function given by :

$$C(d_{ij}) = \frac{1}{\Gamma(\upsilon)2^{\upsilon-1}}(\kappa d_{ij})^\upsilon K_\upsilon(\kappa d_{ij}),$$

where K_υ is the modified Bessel function of second kind and order υ ($\upsilon > 0$), υ is a smoothing parameter controlling the rate of correlation decay fixed to 1, and κ ($\kappa > 0$) is a scaling parameter. The spatial range is defined as the minimum distance at which spatial correlation between locations is less than 10% and is given by $\sqrt{8\upsilon/\kappa}$.

References

Banerjee S, Gelfand AE, Finley AO, and Sang H (2008). Gaussian predictive process models for large spatial data sets. *Journal of the Royal Statistical Society : Series B (Statistical Methodology)*, 70(4) :825–848.

Cameletti M, Lindgren F, Simpson D, and Rue H (2013). Spatio-temporal modeling of particulate matter concentration through the SPDE approach. *AStA Advances in Statistical Analysis*, pp. 1–23.

Chammartin F, Hürlimann E, Raso G, N'Goran EK, Utzinger J, and Vounatsou P (2013a). Statistical methodological issues in mapping historical schistosomiasis survey data. *Acta Tropica*, 128(2) :345–352.

Chammartin F, Scholte RGC, Malone JB, Bavia ME, Nieto P, Utzinger J, and Vounatsou P (2013b). Modelling the geographical distribution of soil-transmitted helminth infections in Bolivia. *Parasites & Vectors*, 6(1) :1–14.

Diggle PJ and Lophaven SÃ (2006). Bayesian geostatistical design. *Scandinavian Journal of Statistics*, 33(1) :53–64.

Johnson ME, Moore LM, and Ylvisaker D (1990). Minimax and maximin distance designs. *Journal of Statistical Planning and Inference*, 26(2) :131–148.

Scheipl F, Fahrmeir L, and Kneib T (2012). Spike-and-slab priors for function selection in structured additive regression models. *Journal of the American Statistical Association*, 107(500) :1518–1532.
